# The Carotenoid Composition of Larvae Feed Is Reflected in Adult House Fly (*Musca domestica*) Body

**DOI:** 10.3390/insects15070521

**Published:** 2024-07-11

**Authors:** Li-Or Lahmi, Ayelet Harari, Aviv Shaish, Ido Tsurim

**Affiliations:** 1 Achva Academic College, Beer-Tuvia Regional Council 7980400, Israel; liorlahmi@gmail.com (L.-O.L.); avivsha30@gmail.com (A.S.); 2 The Bert W. Strassburger Metabolic Center, Sheba Medical Center, Tel-Hashomer, Ramat Gan 5265601, Israel; harari.ayelet1@gmail.com; 3 Katif Center for R&D Coastal Desert, Ministry of Innovation, Science and Technology, Sdot Negev Regional Council, P.O. Box 100, Netivot 8771002, Israel

**Keywords:** substrate, feed, larvae, insects, chemical composition, carotenoids, house fly

## Abstract

**Simple Summary:**

Carotenoids are organic compounds with various important functions in animals. Most animals obtain their carotenoids only from their food. House flies are common pests with a worldwide distribution. Their larvae feed on organic matter that may vary substantially in its carotenoid composition. This study examines if the carotenoid composition in the body of adult house flies is related to the composition in the larval feed. House fly larvae were reared on diets that differed in carotenoid composition. HPLC analysis of the emerging adult flies indicates that the carotenoid composition in adult house flies was similar, but not identical, to the composition in the larval feed. These results suggest that carotenoid composition in adult flies may be used to estimate the composition in their natal habitat and therefore may be used to identify potential sources of house fly infestations. Also, the feed of house fly larvae, used for animal feed, should be carefully considered.

**Abstract:**

Carotenoids are common and diverse organic compounds with various functional roles in animals. Except for certain aphids, mites, and gall midges, all animals only acquire necessary carotenoids through their diet. The house fly (*Musca domestica*) is a cosmopolitan pest insect that populates diverse habitats. Its larvae feed on organic substrates that may vary in carotenoid composition according to their specific content. We hypothesized that the carotenoid composition in the adult house fly’s body would reflect the carotenoid composition in the larval feed. House fly larvae were reared on diets that differed in carotenoid composition. HPLC analysis of the emerging adult flies indicate that the carotenoid composition of adult house flies is related, but not identical, to the carotenoid composition in its natal substrate. These findings may be developed to help identify potential sources of house fly infestations. Also, it is recommended that rearing substrates of house fly larvae, used for animal feed, should be carefully considered.

## 1. Introduction

Carotenoids are common and diverse organic compounds [1]. Some carotenoids have essential functional roles in animals [2], such as in the visual and immune systems, diapause-related processes, protection against oxidative stress, ornament-based signaling, etc. [3,4,5,6]. However, the specific carotenoid composition in most animals and the function and metabolism of most carotenoids are largely unknown. Biosynthesis of carotenoids largely occurs in photosynthetic plants, algae, and some bacteria, archaea, and fungi [7], but it is limited in Animalia to certain aphids, mites, and gall midges [8]. Most animals acquire necessary carotenoids from their diet and use them directly from their food or after modification through metabolic reactions [9,10].

Both diet and genetic background likely affect carotenoid composition in insects’ bodies [11,12]. Holometabolous insects acquire most of the building materials of the adult body from the larval feed during the larval stages. Hence, adult carotenoid body composition and related functions may largely reflect the larvae’s available feed resources [11,12,13]. However, knowledge of the carotenoid pathways between larval feed and adult carotenoid composition and functionality is scant.

The house fly is a cosmopolitan pest insect that poses public health hazards [14] but also possesses growing economic importance in the animal feed industry [15]. The maggot develops in and feeds upon rotting organic substrates, which may differ substantially in their carotenoid composition. Finke et al. [16] reported that adult house flies contained the carotenoids lutein and zeaxanthin but not β-carotene. However, our knowledge of carotenoid function and composition in animals is largely limited to the visual system.

We hypothesized that the carotenoid composition in the adult house fly’s body would reflect its composition in the larval feed (substrate). Hence, the carotenoid composition of adult flies is expected to vary according to differences in the carotenoid composition of the larval feed. Alternatively, if only specific carotenoids can be used for specific biological functions, then unsuitable carotenoids should be modified into specific suitable ones or excreted from the body. The carotenoid composition is then expected to be homogenous among adult flies, even if they developed as larvae on feeds that differed in carotenoid composition.

## 2. Materials and Methods

Four groups of house fly maggots, all originating from the same laboratory colony, were reared simultaneously, each group on a different substrate. Rearing substrates were cucumber, red tomato, yellow tomato, and the control. The latter contained bran and powdered rodent chaw and lacked carotenoids. To establish the four groups, four Petri dishes, 60 mm in diameter and each filled with one of the four substrates, were placed in a cage containing a colony of 5–6 days old house flies, and female oviposition on the substrates was allowed for 24 h. The substrate of each Petri dish, containing 100–200 house fly eggs, was then transferred into a separate rearing cup (90 mm diameter × 65 mm high), containing 300 mL of the same substrate. The cups were then placed in a growing chamber (29 ± 2 °C, 70% humidity, 14:10 dark–light regime) for 10 days. Three 50 mg samples were then taken from each aged substrate type for carotenoid composition analysis, and a layer of dry coarse sawdust was added to allow a suitable environment for pupation. Pupae were collected for five days and kept until hatching in separate, empty containers, plugged with cotton wool, under similar conditions as the larvae. Adult females, identified according to head characteristics, were frozen at −20 °C upon hatching. Substrate samples and five randomly chosen hatched adult females from each substrate type were then analyzed using an untargeted HPLC (Shimadzu, Kyoto, Japan) with a photodiode array detector, as described in [17], to quantify the carotenoid composition in each sample. The limit of detection (LOD) was 0.01 µg/g. The limit of quantitation was approximately 0.05 µg/g and detected carotenoids below this level were noted as ‘detected’ (Table 1) but were not quantified. As a byproduct, this methodology also provided the chlorophyll content of the samples. For full details and the structure of all carotenoids described in this study, consult the *Carotenoid Handbook* [1].

## 3. Results

**Figure 1:**
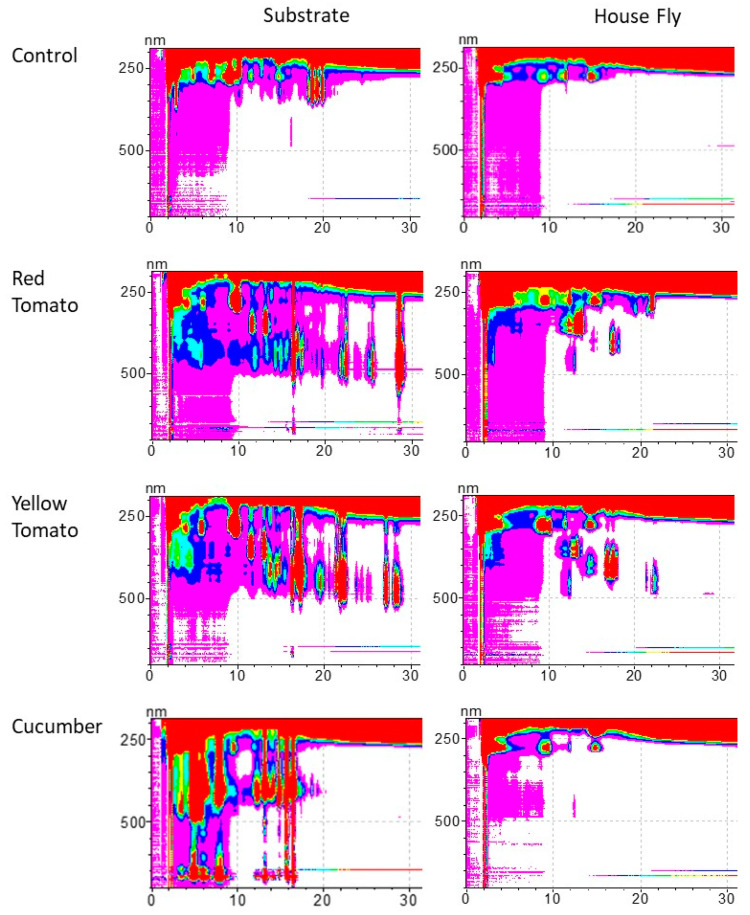
HPLC analysis of the carotenoids in substrate and adult flies. The figure displays one representative contour plot from the substrates and the respective adult flies. Carotenoids were identified according to their characteristic retention time (Rt) and absorbance. Phytoene (Rt 5.8, 6.4, 9.9 min), phytofluene (Rt 11.7, 12.4, 13.2 min), ζ-carotene (Rt 16.9, 17.1, 17.9 min), asymmetric ζ-carotene (Rt 14.4, 14.8 min), neurosporene (Rt 21.4, 22.3 min), lycopene (Rt 21.7, 22.3, 24.9, 25.2, 28.5 min), prolycopene (Rt 17.3), and lutein (Rt 5.8).

The HPLC analysis indicates that the four substrates differed substantially in their carotenoid composition (Table 1). The carotenoid analysis of the respective adult house flies indicates that adult house flies that were reared on different larval substrate types differed in their carotenoid composition. However, the carotenoid composition in the adult flies was not identical to the carotenoid composition in the substrate. Adult flies only contained carotenoids in the substrate, but not all the carotenoids in the substrate were found in the adults. Moreover, lycopene and prolycopene, the commonest carotenoids in the red and yellow tomato substrates, respectively, were completely absent from the adult flies (Table 1, Figure 1).

Control: As expected, the control substrate did not contain carotenoids. Accordingly, carotenoids were not detected in the emerging adult flies.

Cucumber: This substrate did not contain carotenoids. Accordingly, the adult house flies emerging from this substrate did not contain carotenoids. While this substrate contained chlorophylls, the adults emerging from it did not contain this pigment.

Red tomato: This substrate contained several carotenoids: phytoene, two isomers of phytofluene, β-carotene, lutein, asymmetric ζ-carotene, 3 isomers of ζ-carotene, and several isomers of lycopene, which was the dominant carotenoid. Surprisingly, the adult house flies emerging from this substrate did not contain lycopene, phytoene, β-carotene, and lutein. Even so, the adult flies did contain phytofluene, asymmetric ζ-carotene, and ζ-carotene, which were of similar isomers to those found in the substrate.

Yellow tomato: The yellow tomato substrate contained phytoene, two isomers of phytofluene, β-carotene, asymmetric ζ-carotene, 3 isomers of ζ-carotene, and several isomers of lycopene. Unlike the red tomato substrate, the primary carotenoids in the substrate were prolycopene and neurosporene. Interestingly, the adult house flies emerging from this substrate did not contain prolycopene and lycopene, but, similarly to adult flies from the red tomato substrate, contained isomers of phytofluene and ζ-carotene. In addition, adult flies also contained neurosporene.

## 4. Discussion

This study shows that the carotenoid composition in adult house flies is related to the carotenoid composition in larval feed. These findings support the primary hypothesis and agree with previous findings, e.g., [11] on silkworms and [12] on dragonflies. However, the carotenoid composition in the adult flies was not identical to the carotenoid composition in the respective larval feed. Interestingly, phytoene, β-carotene, lutein, prolycopene, and lycopene were absent from adult flies originating from the substrates containing fair amounts of these carotenoids. It is possible that these carotenoids were not absorbed by the larvae from the feed. However, a more interesting possibility is that they were absorbed and subsequently metabolized into other compounds. These findings strongly suggest that house flies, and likely other insects, selectively absorb and/or metabolize carotenoids from their feed [11]. As far as we know, the presence of the carotenoids phytofluene, ζ-carotene, asymmetric ζ-carotene, and neurosporene has not been described before in house flies.

In agreement with Finke et al. [16], we did not find β-carotene in the adult flies. However, Finke et al. [16] reported zeaxanthin and lutein, which were not found in the adult house flies in the present study. Indeed, zeaxanthin was absent from all substrates in the present study, and lutein was present only in very small amounts in the red tomato substrate. 

The specific function of the carotenoids detected in the house flies, and their metabolic pathways, are largely unknown. Further work is required to unravel the role of these carotenoids in the biology and ecology of house flies (and other insects), the consequences of habitat variability on carotenoid composition, and the biochemical pathways of carotenoid absorption and metabolism. Interestingly, this study indicates that the carotenoid composition of adult house flies reflects substrate-specific carotenoid fingerprints. This relationship could potentially be used to identify the natal habitats of free-ranging adult house flies, possibly aiding in identifying and locating sources of house fly infestation. The use of insects, including house flies, has increasingly been promoted as a replacement for other animal feed sources, especially for fish and poultry [18,19]. Considering the findings of this study, the specific composition of house fly feed should be considered not only with respect to protein and fat content but also to other essential compounds, such as vitamins and carotenoids.

## Figures and Tables

**Table 1:** Carotenoid content in the feed (3 replicates for each type; ng/g ± 1 se) and in the adult house flies (5 flies from each substrate type; ng/fly ± 1 se). Different isomers of each carotenoid type are summed together. The limit of detection (LOD) was 0.01 µg/g. The limit of quantitation was approximately 0.05 µg/g; detected carotenoids below this level are noted as ‘detected’.

Carotenoid	Substrate Category	Carotenoids in the Substrate (µg/g)	Carotenoids in the Adult Fly (ng/fly)
Phytoene	Red Tomato Yellow Tomato Cucumber Control	12.8 ± 2.7 33.5 ± 3.3 <LOD <LOD	<LOD <LOD <LOD <LOD
Phytofluene	Red Tomato Yellow Tomato Cucumber Control	3.1 ± 0.7 7.8 ± 1.1 <LOD <LOD	323.0 ± 248.9 88.1 ± 43.3 <LOD <LOD
Lutein	Red Tomato Yellow Tomato Cucumber Control	0.1 ± 0.03 <LOD <LOD <LOD	<LOD <LOD <LOD <LOD
Asymmetric ζ-carotene	Red Tomato Yellow Tomato Cucumber Control	Detected 0.6 ± 0.1 <LOD <LOD	13.8 ± 6.0 22.2 ± 11.0 <LOD <LOD
ζ-carotene	Red Tomato Yellow Tomato Cucumber Control	0.5 ± 0.1 Detected <LOD <LOD	71.6 ± 35.6 373.5 ± 347.5 <LOD <LOD
β-carotene	Red Tomato Yellow Tomato Cucumber Control	4.1 ± 0.5 1.4 ± 0.6 <LOD <LOD	<LOD <LOD <LOD <LOD
Prolycopene	Red Tomato Yellow Tomato Cucumber Control	<LOD 9.2 ± 1.8 <LOD <LOD	<LOD <LOD <LOD <LOD
Neurosporene	Red Tomato Yellow Tomato Cucumber Control	<LOD 1.8 ± 0.3 <LOD <LOD	<LOD 16.4 ± 17.9 <LOD <LOD
Lycopene	Red Tomato Yellow Tomato Cucumber Control	13.8 ± 4.2 0.9 ± 0.3 <LOD <LOD	<LOD <LOD <LOD <LOD

## Data Availability

The data presented in this study are available on request from the corresponding author.
